# Cucurbiturils in Oxygen Delivery and Their Potential in Anemia Management

**DOI:** 10.3390/jcm14238571

**Published:** 2025-12-03

**Authors:** Daniel Papiu, Alexandra Nadaban, Adelina Palcu, Alciona Sasu, Gabriela Mara, Paul Albu, Casiana Boru, Coralia Cotoraci

**Affiliations:** 1 Multidisciplinary Doctoral School, Vasile Goldis Western University of Arad, 310414 Arad, Romania; papiu.daniel@uvvg.ro; 2 Department of Hematology, Faculty of Medicine, Vasile Goldis Western University of Arad, 310414 Arad, Romania; nadaban.alexandra@uvvg.ro (A.N.); palcu.adelina@uvvg.ro (A.P.); sasu.alciona@uvvg.ro (A.S.); cotoraci.coralia@uvvg.ro (C.C.); 3 Pneumology Department, Vasile Goldis Western University of Arad, 310414 Arad, Romania; mara.gabriela@uvvg.ro; 4 Department of Pharmaceutical Sciences, Faculty of Pharmacy, Vasile Goldis Western University of Arad, 86 L. Rebreanu St., 310414 Arad, Romania; 5 Department of Obstetrics and Gynecology, Faculty of Medicine, Vasile Goldis Western University of Arad, 310414 Arad, Romania

**Keywords:** cucurbiturils, supramolecular chemistry, oxygen carriers, anemia diagnostics and therapy, biocompatibility

## Abstract

Efficient oxygen transport and accurate anemia diagnostics remain significant medical challenges, as current therapies suffer from stability limitations, immunogenic risks, and inadequate sensitivity. Cucurbiturils (CB[n]), a family of pumpkin-shaped supramolecular macrocycles, present promising solutions due to their rigid architecture, hydrophobic cavities, and strong host–guest binding properties. Functional derivatives such as perhydroxy-cucurbit[5]uril can reversibly bind dioxygen under physiological conditions, highlighting their potential as synthetic hemoglobin substitutes. Additionally, cucurbituril-based probes for Fe^3+^ and folate enable sensitive and selective detection of iron- and folate-deficiency anemia. Biocompatibility assessments in vitro and in vivo indicate low systemic toxicity and acceptable hemocompatibility for homologues such as CB[6], CB[7], and CB[8], though apoptosis, myotoxicity, or cardiotoxicity may occur at elevated concentrations. These data emphasize the need for thorough toxicological evaluation but also demonstrate that cucurbiturils provide a versatile platform for oxygen transport, diagnostic applications, and drug-delivery strategies in anemia management. While their translation to clinical practice is still at an early stage, the structural tunability, stability, and encouraging safety profile of CB[n] macrocycles offer a strong basis for continued biomedical development.

## 1. Introduction

Efficient oxygen delivery and accurate hematological diagnostics are vital to human health, yet current solutions—such as blood transfusions, hemoglobin-based oxygen carriers, semisynthetic and synthetic red blood cell (RBC) surrogates in cross-linked, polymeric, or encapsulated forms, and conventional iron assays—are limited by stability issues, immunogenic risks, and diagnostic delays [1].

Artificial oxygen carriers are being developed as substitutes for red blood cells in severe blood loss and critical care settings. They offer therapeutic oxygen delivery when transfusion is limited, with applications in trauma, surgery, hemorrhagic shock, stroke, myocardial infarction, and sickle cell crisis. Current approaches include hemoglobin-based, perfluorocarbon-based, stem cell-derived, and micro/nanobubble systems [2]. Despite challenges related to stability, toxicity, and immune compatibility, artificial oxygen carriers show significant potential.

Supramolecular chemistry offers a novel path forward, and cucurbiturils (CB[n]) have emerged as exceptional candidates. These rigid, pumpkin-shaped macrocycles combine high binding affinity, remarkable stability, and biocompatibility, enabling precise host–guest interactions with small gases, metal ions, and biomolecules.

Functional derivatives such as perhydroxy-cucurbit[5]uril have demonstrated the ability to reversibly bind molecular oxygen under physiological conditions, opening prospects for synthetic oxygen carriers in conditions of hypoxia or anemia [3]. In parallel, cucurbituril-based probes for Fe^3+^ detection and folate inclusion complexes offer sensitive, rapid, and selective tools for diagnosing and potentially treating iron- and folate-deficiency anemia [4,5].

This review aims to provide a comprehensive analysis of cucurbituril-based systems with relevance to oxygen transport and hematology, emphasizing their structural features, binding mechanisms, and emerging biomedical applications. Because this work is a narrative review, all findings discussed herein derive exclusively from secondary sources, as no original experiments were performed. Particular focus is placed on their potential as synthetic oxygen carriers and as diagnostic/therapeutic tools for anemia, highlighting current achievements, limitations, and future research directions toward clinical translation.

## 2. Cucurbiturils—Structure and Properties

### 2.1. Discovery of Cucurbiturils: Early Studies and Applications

Cucurbiturils are a comparatively recent class of molecular containers that form stable host–guest complexes with diverse species, including drugs, amino acids, peptides, saccharides, dyes, hydrocarbons, and proteins [1,6]. These supramolecular macrocycles consist of glycoluril units (=C_4_H_2_N_4_O_2_=) linked by methylene (-CH_2_-) bridges.

The first cucurbituril-type product was described in 1904 by Eberhardt Meyer as the condensation product of glycoluril and formaldehyde [2,7]. In 1905, Behrend and co-workers reported a related cross-linked polymer that, upon treatment with concentrated H_2_SO_4_, afforded a crystalline precipitate [3,8]. Lacking access to X-ray crystallography, the molecular structure remained unresolved for decades.

In 1981, Mock and Freeman (University of Illinois) reproduced the Meyer–Behrend reaction, isolating a crystalline macrocycle composed of six glycoluril units bridged by twelve methylene linkers [7,8,9]. The compound’s pumpkin-like morphology inspired the name cucurbituril, after the Cucurbitaceae plant family.

Freeman subsequently determined the crystal structure of the first cucurbit[6]uril (CB[6]) host–guest complex, encapsulating the p-xylylenediammonium cation [8]. Early investigations by Mock demonstrated that cucurbit[6]uril (CB[6]) is able to form strong 1:1 inclusion complexes with a variety of alkyl- and diammonium ions in aqueous formic acid solutions [10]. Subsequent studies expanded on these findings, highlighting the ability of CB[6] to exploit its confined hydrophobic cavity for catalysis and to serve as a platform for the development of switchable pseudorotaxane architectures [11].

Renewed interest in crystal engineering and noncovalent chemistry during the 1980s–1990s highlighted cucurbiturils as versatile mediators of supramolecular interactions [11]. Between 2000 and 2002, Kim and co-workers refined the classical Meyer–Behrend reaction, which enabled the selective preparation and crystallization of cucurbit[5]uril, cucurbit[7]uril, and cucurbit[8]uril [12]. In 2001, Day and co-workers reported the presence of cucurbit[10]uril (CB[10]) as part of a CB[5]–CB[10] complex [13], and in 2005 Lagona and colleagues succeeded in isolating pure CB[10] [14]. These cucurbituril homologues consist of 5, 6, 7, 8, or 10 glycoluril units, respectively. Lee et al. also investigated the host–guest chemistry of CB[6] in neutral aqueous solution, which significantly broadened its applicability for constructing complex supramolecular architectures, including so-called “molecular necklaces [15].

### 2.2. Molecular Structure and Key Features of Cucurbiturils

Cucurbiturils (CB[n]) are a family of pumpkin-shaped macrocyclic hosts characterized by a rigid hydrophobic cavity and two identical carbonyl-lined portals that enable selective molecular recognition [11]. These macrocycles are composed of *n* glycoluril units interconnected by methylene bridges, with the ring size directly dictating both the portal diameter and the internal cavity volume [12]. The hydrophobic interior accommodates nonpolar guests, while the polar portals bind cations via ion–dipole and hydrogen-bond interactions [5]. Extensive hydrogen bonding interactions between the carbonyl portals and encapsulated guests, as well as among the glycoluril units themselves, play a pivotal role in maintaining the structural integrity and stability of cucurbituril host–guest systems [11,12].

Among the cucurbituril homologues, the most extensively studied are CB[5] (C_30_H_30_N_20_O_10_), CB[6] (C_36_H_36_N_24_O_12_), and CB[7] (C_42_H_42_N_28_O_14_) [16]. CB[5], first reported by Kim and co-workers in 2000, features a portal diameter of 2.4 Å and a cavity volume of 82 Å^3^—less than half that of CB[6]—which restricts the encapsulation of bulky guests [12]. Nevertheless, its carbonyl-lined portals readily bind cations such as alkali, alkaline-earth, and ammonium ions, as well as small molecules like hexamethylenetetramine [7]. Furthermore, CB[5] and its decamethylated derivative are capable of encapsulating small gases (N_2_, O_2_, Ar, CO_2_, etc.) in both the solid state and in aqueous solution [11].

Larger homologues offer greater versatility: CB[7] forms strong 1:1 binary complexes, while CB[8] accommodates heteroternary complexes with aromatic compounds [2]. Guest species range from small organics and amino acids to peptides and proteins [17]. Binding is driven by hydrophobic effects and release of high-energy water, with optimal guest volume at ~55% of the host cavity. CB[7] exhibits some of the highest known binding affinities, surpassing even biotin–avidin [17].

Larger cucurbituril homologues offer enhanced versatility: cucurbit[7]uril (CB[7]) forms strong 1:1 binary complexes, whereas cucurbit[8]uril (CB[8]) can host heteroternary complexes with aromatic compounds [15]. Guest species range from small organic molecules and amino acids to peptides and proteins [7]. The binding process is mainly driven by hydrophobic effects and the release of high-energy water, with an optimal guest volume corresponding to ~55% of the host cavity. Notably, CB[7] exhibits some of the highest binding affinities reported to date, exceeding even the biotin–avidin interaction [7].

Key properties of cucurbiturils include high thermal/chemical stability, tunable cavity sizes, biocompatibility, and exceptionally strong host–guest binding—often orders of magnitude greater than cyclodextrins in water [17]. In drug delivery, CB[7] coatings can slow release at physiological pH [7]. Solid-state complexes are prepared by stoichiometric mixing followed by lyophilization or ball milling [7].

**Figure 1:**
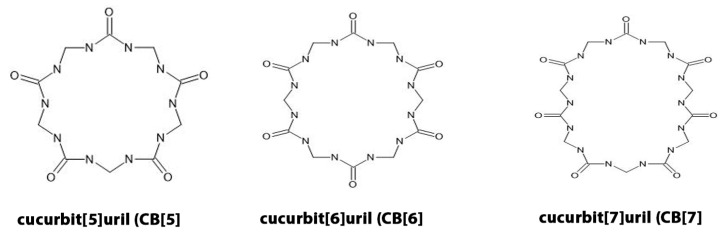
Chemical structures of representative cucurbituril homologues.

The chemical structures of representative cucurbituril homologues (CB[5], CB[6], and CB[7]) are shown in Figure 1, illustrating the cyclic glycoluril framework and the carbonyl portals involved in host–guest interactions.

### 2.3. Physicochemical Properties and Relevance for Oxygen Transport

The solubility of cucurbiturils (CB[n]) and their complexes varies significantly across the homologous series [7]. CB[5] and CB[7] are considerably more water-soluble than CB[6] and CB[8], with solubility influenced by solvent, temperature, ionic strength, and guest identity [7,17]. Low aqueous solubility can be enhanced by the presence of cations or positively charged guests [11]. CB[7] exhibits sequence-specific molecular recognition applicable in detection and separation processes [7], making cucurbiturils attractive not only as synthetic receptors but also as scaffolds for supramolecular architectures [14].

Key features include [11]:

Recognition of cationic and neutral guests in aqueous media;

Cavity volumes ranging from 68 to 691 Å^3^;

Guest selectivity determined by cavity size, from small gases (CB[5]) to bulky aromatic/polycyclic species (CB[7]) and dual complementary guests (CB[8]).

Gas absorption is generally favored for smaller homologues, as CB[5] and CB[6] cavities better accommodate small molecules than CB[7] or CB[8] (2). CB[5] is suited for aqueous-phase gas uptake, whereas less soluble CB[6] can be used in powder form for flue gas cleaning [7]. Permethylated CB[5] encapsulates a variety of gases in both solid and aqueous phases, with N_2_O and CO_2_ showing the highest affinity. Larger gases such as Xe or CH_4_ require heating (~80 °C) for encapsulation in aqueous solution, suggesting a role of solvent in guest inclusion [7].

CB[n] homologues have also been oxidized to perhydroxy derivatives, which can degrade to oxalic acid, demonstrating chemical reactivity under oxidative conditions [17]. Cellular permeability studies indicate that CB[7] and CB[8] complexes can cross murine macrophage membranes and remain intact for up to 2 h [7].

In oxygen transport contexts, both aqueous solubility and the ability to bind small neutral gases are critical [3]. Native CB[5] and CB[6] exhibit very low solubility (~0.3 mM and ~0.02 mM, respectively, at 293 K), but functionalization can dramatically improve this. For example, decamethyl-CB[5] is 27× more soluble than native CB[5], and perhydroxy-CB[5] reaches ≥0.7 mM solubility at 293 K [3]. Gas molecules occupy the hydrophobic cavity, while cations bind at portals; no significant anion binding in pure water has been reported [3]. Potassium and ammonium salts show high affinity for perhydroxy-CB[5] compared with native or permethylated analogues [3].

## 3. Mechanism of Action of Cucurbiturils in Oxygen Transport

Oxygen delivery is central to human physiology, and deficiencies in oxygen transport—whether due to acute hemorrhage, chronic anemia, or ischemic conditions—are associated with high morbidity and mortality worldwide [17,18]. Current solutions, such as red blood cell transfusions [19], hemoglobin-based oxygen carriers [20], and perfluorocarbon emulsions [21], suffer from limitations including storage instability, short shelf life, immunogenic reactions, oxidative side effects, and restricted availability. These challenges underscore the need for alternative molecular platforms capable of binding and releasing O_2_ reversibly under physiological conditions, while maintaining safety and stability profiles compatible with clinical use.

Supramolecular chemistry offers unique opportunities in this regard. Among the promising candidates, cucurbiturils (CB[n])—rigid, pumpkin-shaped macrocyclic hosts—have emerged as versatile scaffolds due to their ability to form stable host–guest complexes with small neutral gases, ions, and biomolecules [22].

Cucurbiturils are composed of glycoluril units linked by methylene bridges, forming a hydrophobic cavity with two carbonyl-lined portals [23]. The size of the cavity is determined by the number of glycoluril units (n = 5, 6, 7, 8, or 10), enabling selective inclusion of guest molecules [22]. CB[5], in particular, offers an internal volume suitable for small diatomic gases such as O_2_ and N_2_ [3]. The hydrophobic interior stabilizes these neutral species through van der Waals interactions, while the portals interact with cations, enhancing complex stability [14].

Earlier studies demonstrated that solid-state and solution-phase CB derivatives could encapsulate small gases, but the biomedical relevance of these findings remained speculative until functionalized derivatives—especially perhydroxy-cucurbit[5]uril (CB[5]-(OH)_10_)—were introduced [13,24].

Binding studies revealed that among three cucurbit[5]uril derivatives tested, only the per-hydroxylated form ((OH)10-CB[5]) could significantly encapsulate dioxygen at physiological temperature, with stability retained even in saline conditions mimicking blood, thereby highlighting its potential as a supramolecular oxygen carrier in haemoglobin substitute formulations [3].

**Figure 2:**
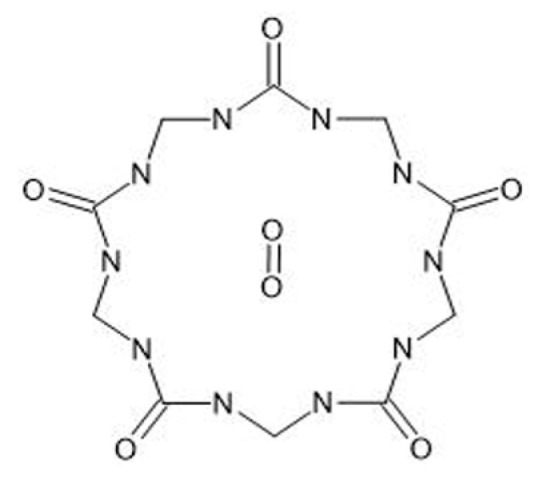
Chemical structure of perhydroxy-cucurbit[5]uril (PHCB[5]. Created with ChemDraw 25.0).

To complement the discussion on oxygen encapsulation, Figure 2 shows the chemical structure of perhydroxy-cucurbit[5]uril (PHCB[5]) and the proposed binding site for molecular oxygen within its hydrophobic cavity. The hydrophilic hydroxyl substituents improve solubility, while the internal nonpolar pocket enables reversible O_2_ entrapment through van der Waals interactions. This supramolecular configuration supports the hypothesis that PHCB[5] can act as a synthetic oxygen carrier under physiological conditions.

Unlike hemoglobin, cucurbituril-based oxygen carriers lack a central metal coordination site and therefore exhibit negligible affinity for carbon monoxide (CO). While native cucurbiturils can weakly encapsulate small neutral gases such as CO, N_2_, and O_2_ through van der Waals interactions in the solid state or aqueous media, no strong coordination comparable to the heme–CO complex has been reported. This absence of competitive CO binding may confer a theoretical advantage for oxygen delivery under CO-rich or hypoxic conditions, as cucurbituril–oxygen complexes would not be displaced by CO in the manner observed with hemoglobin. Notably, perhydroxy-cucurbit[5]uril retains stable O_2_ binding under physiological saline conditions, and current evidence suggests that carbon monoxide does not interfere with this interaction. Although this observation supports the conceptual feasibility of cucurbituril-based carriers in scenarios such as carbon monoxide poisoning, experimental validation under controlled CO exposure remains necessary to confirm their oxygen transport efficiency and safety in vivo [3,7,24].

In biological oxygen transport, O_2_ binds reversibly to the ferrous (Fe^2+^) center of the heme group in hemoglobin, not to ferric (Fe^3+^) iron. The resulting Fe^2+^–O_2_ complex exhibits partial electron transfer between Fe^2+^ and O_2_, generating a resonance hybrid between Fe^2+^–O_2_ and Fe^3+^–O_2_^−^ states. This reversible redox-like process is distinct from the irreversible oxidation of Fe^2+^ to Fe^3+^ observed in methemoglobin, which is unable to bind O_2_. This clarification has been included to ensure accuracy in describing oxygen binding mechanisms and to prevent confusion with the ferric (Fe^3+^) state represented in the earlier figure.

### 3.1. Stability of the Cucurbituril–Oxygen Complex

The stability of cucurbituril–oxygen complexes was investigated [3]. Building on the observation that decamethyl-cucurbit[5]uril (DMCB[5]) binds oxygen in the gas phase, in pure water, and in the solid state, the study examined O_2_ binding in aqueous solutions, both in the absence and presence of cations, for CB[5], DMCB[5], and perhydroxy-cucurbit[5]uril (PHCB[5]). CB[5] showed negligible oxygen affinity, whereas both DMCB[5] and PHCB[5] bound O_2_ in pure water. Notably, DMCB[5] could bind oxygen without added cations [3].

Subsequent experiments evaluated O_2_ binding under physiological conditions (310 K, 0.15 M NaCl). In these conditions, the high Na^+^ concentration—comparable to that in blood—precluded DMCB[5] from serving as an oxygen carrier. By contrast, PHCB[5] maintained significant oxygen-binding capacity, making it a promising candidate for biomedical O_2_ delivery [3].

Based on the experimentally determined O_2_ binding constant for PHCB[5], the solubility of O_2_ in saline, and the fraction of host molecules available for binding, it was estimated that a concentration of ~20–30 mM PHCB[5] would be required to transport oxygen at physiological blood levels (~7.4 mM O_2_, equivalent to 190 mL O_2_·L^−1^) [3,12].

### 3.2. Biocompatibility of Cucurbiturils—Relevant Preclinical and Clinical Studies

Biocompatibility assessments of cucurbiturils have been carried out in both in vitro and in vivo models. findings suggest that, although certain cucurbiturils may induce early apoptotic effects in lymphocytes and CB[7] can enhance hemolysis under physiological conditions, the overall low cytotoxicity and acceptable hemocompatibility profile observed up to concentrations of 0.3 mM support their further development as modifiable supramolecular scaffolds for clinically relevant drug delivery systems [25]. Other studies demonstrate that cucurbit[7]uril and cucurbit[8]uril are generally well tolerated, with in vitro experiments showing an IC_50_ of about 0.53 mM for CB[7] and no significant cytotoxicity for CB[8] within its solubility range; in vivo investigations in mice further confirmed this safety profile, reporting tolerance up to 250 mg kg^−1^ for intravenous CB[7] administration and up to 600 mg kg^−1^ for oral administration of a CB[7]/CB[8] mixture [26]. Other findings further support this view, showing that while CB[6], CB[7], and linear derivatives such as Motor2 may exhibit some myotoxic and cardiotoxic effects in ex vivo assays, they lack measurable neurotoxicity and, importantly, CB[7] can markedly reduce the myo- and cardiotoxicity of cisplatin upon encapsulation, consistent with the overall low toxicity profile of cucurbiturils observed in systemic studies [27]. Other data obtained from zebrafish models thus corroborate the generally favorable safety profile of CB[7], indicating that while cardiotoxic and behavioral effects become apparent only at higher micromolar concentrations (≥500 μM), developmental and hepatic toxicities remain negligible at sub-toxic levels, thereby reinforcing its suitability for further biomedical investigation [28].

Toxicological and bioactivity assessments of CB5, CB7, and several acyclic CB analogs revealed no toxicity at the relevant concentrations [29].

According to Kim et al., CB molecules are non-toxic toward human lung and ovarian cancer cells, showing ED_50_ values greater than 100 μM [30].

Other data from in vivo studies further highlight the high tolerability of CB[7], showing that single administrations at oral doses up to 5 g/kg, peritoneal doses up to 500 mg/kg, and intravenous doses up to 150 mg/kg did not cause measurable changes in body weight, hematological indices, or hepatic and renal biochemical markers over a 21-day observation period; moreover, histopathological analyses of the heart, liver, spleen, lungs, kidneys, and gastrointestinal tissues revealed no evidence of structural damage or inflammatory infiltration, thereby underscoring the low systemic toxicity of CB[7] and supporting its consideration as a safe supramolecular platform for further biomedical exploration [31].

To assess the cytotoxic potential of different cucurbiturils, CB[5], CB[6], and CB[7] were tested on HaCaT keratinocytes and erythrocytes. The results showed that CB[5] and CB[6] exhibited no cytotoxicity even at concentrations as high as 30 mg/mL, whereas CB[7] induced apoptosis at 3.75 mg/mL. Importantly, none of the cucurbiturils investigated caused hemolysis in erythrocytes, which support the favorable safety profile of cucurbiturils and highlight their suitability as host–guest complexes for biomedical applications [32].

### 3.3. Applicability of Cucurbiturils in Anemia Treatment—Current Status and Perspectives

**Figure 3:**
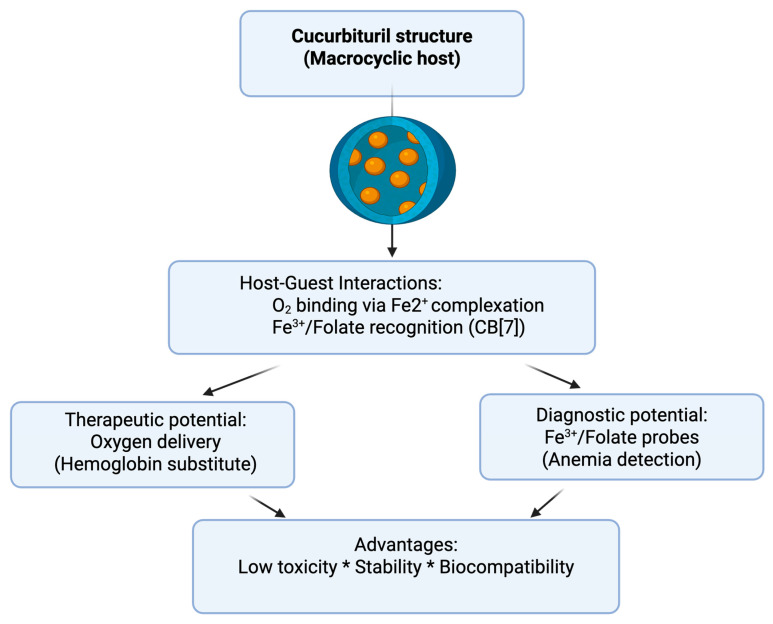
Cucurbiturils in oxygen delivery/anemia management. Created with Biorender.com.

The therapeutic use of cucurbiturils in anemia is still at a conceptual stage, with most research focusing on exploratory studies rather than clinical application. Nevertheless, recent findings highlight their potential for diagnostic and therapeutic roles in anemia-related conditions (Figure 3).

Iron deficiency remains one of the most prevalent causes of anemia. Iron (Fe), a key transition metal, is essential for oxygen transport, cellular respiration, and numerous enzymatic processes. Deviations from normal Fe^3+^ levels—whether deficiency or overload—can lead to severe disorders including anemia, cardiomyopathy, and cancer [33]. Precise detection of Fe^3+^ in living cells is thus critical for early diagnosis and treatment planning. A 2023 study introduced an acyclic cucurbituril-based fluorescent probe capable of detecting Fe^3+^ in live cells with high sensitivity and operational simplicity, offering advantages over conventional quantitative assays and marking an important step toward modern, rapid iron deficiency diagnostics [33].

Folate deficiency is another significant contributor to anemia, particularly megaloblastic anemia. Folate is crucial for amino acid metabolism and the synthesis of purines and pyrimidines. Its deficiency has been directly linked to neural tube defects, certain cancers, and other metabolic disorders [4]. In 2021, a supramolecular inclusion complex between cucurbit[7]uril and folate was characterized in a 2:1 stoichiometry using fluorescence spectroscopy, UV–Vis absorption, NMR, and molecular modeling. This complex enabled direct fluorescence detection of folate in biological fluids and provides a foundation for targeted delivery of folate antagonists—an approach with potential relevance for both anemia diagnostics and targeted cancer therapy [4].

In addition to diagnostic roles, cucurbiturils—specifically perhydroxy-cucurbit[5]uril—are under investigation as synthetic oxygen carriers for use under physiological conditions. This derivative has demonstrated the ability to bind and release oxygen at 37 °C in saline concentrations comparable to blood, suggesting its possible role as a hemoglobin substitute [34].

Beyond their conceptual role as supramolecular oxygen carriers, cucurbiturils may contribute to anemia management through both therapeutic and diagnostic mechanisms. Functional derivatives such as perhydroxy-cucurbit[5]uril (PHCB[5]) have been shown to reversibly bind dioxygen under physiological conditions (37 °C, 0.15 M NaCl), maintaining complex stability in saline solutions that mimic blood plasma [3]. This finding supports their potential application as synthetic oxygen carriers, offering a stable and non-immunogenic alternative to hemoglobin-based systems.

In addition, supramolecular probes based on cucurbituril scaffolds have demonstrated high selectivity for anemia-related biomarkers. Acyclic cucurbituril derivatives can sensitively detect Fe^3+^ ions in living cells [33], while CB[7]–folate inclusion complexes have been used for fluorescence-based folate detection and targeted drug delivery [4]. Such diagnostic approaches may complement current iron and folate assays, enabling faster and more selective detection of nutritional and hematological deficiencies.

Regarding biocompatibility and circulation stability, preclinical studies have consistently shown low systemic toxicity and good hemocompatibility. CB[7] and CB[8] were well tolerated in mice, with intravenous doses up to 250 mg·kg^−1^ and oral doses up to 600 mg·kg^−1^ showing no changes in hematological, hepatic, or renal markers [26,31]. Zebrafish models similarly confirmed the absence of developmental and hepatic toxicity at sub-millimolar concentrations [28].

Pharmacokinetic data indicate that smaller homologues (CB[5], CB[6]) are primarily cleared via renal excretion, while larger ones (CB[7], CB[8]) exhibit longer circulation times before urinary elimination, remaining chemically intact during excretion [29]. No metabolic degradation or tissue accumulation has been reported.

In addition, recent studies have highlighted the role of ferroptosis, a regulated, iron-dependent form of non-apoptotic cell death characterized by lipid peroxidation and accumulation of reactive oxygen species [35]. Dysregulated Fe^2+^/Fe^3+^ balance and oxidative stress promote ferroptotic mechanisms that contribute to anemia, tissue injury, and inflammation [36]. Given the high selectivity of cucurbiturils for Fe^3+^ ions, these macrocycles may potentially modulate iron homeostasis and mitigate ferroptosis-related oxidative damage, an aspect that warrants further investigation in future studies.

To date, no clinical trials have evaluated cucurbiturils for anemia or oxygen-transport therapies, and their translation to medical use remains at a preclinical stage [34]. Nevertheless, their chemical inertness, oxygen-binding reversibility, and favorable biocompatibility profile support the continued development of cucurbituril-based materials as potential supramolecular platforms for oxygen delivery and anemia diagnostics.

## 4. Advantages of Cucurbituril Applications and Future Research Directions

### 4.1. Benefits of Cucurbiturils Compared with Other Therapeutic Approaches

Due to their unique molecular recognition properties and very low cytotoxicity, cucurbiturils have attracted significant interest in medicine, particularly for stable drug formulation, targeted delivery, controlled release, and bioanalytical detection of various compounds [34].

Cucurbiturils form stable host–guest complexes with a wide range of therapeutic agents, including antineoplastics, antimicrobial agents, neurotransmitters, neuromuscular blockers, local anesthetics, vitamins, hormones, enzyme inhibitors, and antituberculosis drugs. Complexation often enhances the pharmacological performance of the drug. For example, complexation of the fungicide carboxin with cucurbit[8]uril significantly increased its efficacy, as evidenced by greater inhibition of *Rhizoctonia solani* mycelial growth [17].

For antineoplastic agents, cucurbituril complexation can reduce drug toxicity and, in some cases, enhance antitumor activity [37]. Similarly, stable complexes between cucurbit[7]uril and ranitidine—an H_2_ receptor antagonist used for gastric ulcer treatment—have been shown to increase the drug’s thermal stability [38].

In the case of antibiotics, cucurbit[7]uril can enhance antibacterial activity through complexation, as observed for norfloxacin and ofloxacin [39].

Drug release from cucurbituril complexes generally occurs with rapid kinetics, with association and dissociation rate constants in the order of seconds or faster, resulting in a dynamic equilibrium favorable for drug delivery [40]. Dilution, such as that that occurs in body fluids, can trigger spontaneous release. However, some cucurbiturils allow slower release over hours—e.g., cucurbit[10]uril in ruthenium complexes—making them suitable for sustained-release applications [41,42].

Release can also be triggered by environmental changes, including pH shifts or light exposure [43]. Targeted delivery is feasible, with studies primarily focusing on cucurbit[6]uril-based systems [17].

Beyond enhancing efficacy, cucurbiturils can attenuate and modulate side effects. In zebrafish and mouse models, cucurbit[7]uril inhibited seizures induced by small toxic molecules, demonstrating its potential as an efficient detoxifying agent [17].

### 4.2. Potential Biomedical Applications of Cucurbiturils

Over 30 cucurbituril derivatives are currently known. Since native cucurbiturils are chemically inert, derivatization is a key strategy to enhance their functionality and expand their biomedical applications. The most relevant modifications for medical use have been developed for CB[5], CB[6], CB[7], and CB[8] [34]. One study showed that per-hydroxylated cucurbit[5]uril is capable of binding oxygen, suggesting potential use as a hemoglobin substitute [3].

In CB[6], mono- and perfunctionalized derivatives have been explored for the production of macrocyclic vesicles with diverse applications in therapy and bioimaging, particularly as broadly applicable nanocapsules [34]. CB[6] derivatives have also been studied for tissue engineering. Kim and collaborators developed supramolecular hydrogels that mimic the three-dimensional organization of living tissue, successfully guiding controlled chondrogenesis of human mesenchymal stem cells for potential use as cartilage regeneration scaffolds [44]. The same group designed CB[6]-based sensors capable of selectively detecting acetylcholine in the presence of choline [45].

CB[7] has a wide range of biomedical prospects, including targeted delivery in cellular systems; functionalization as CB[7]–ADA or CB[7]–Fc to link polymer beads, proteins, DNA chains, mitochondria, or even entire cells; fluorescent CB[7] for protein localization in living cells, membrane fusion monitoring, deep-tissue imaging, quantitative DNA analysis, and detection of bioorganic markers; drug delivery systems; applications in nanomedicine and gene therapy; cell-surface regulation for use in cell biology; molecular motors based on CB[7] [17,22,34,46].

Compared to smaller homologues, CB[8] has a larger cavity, enabling it to bind bulkier molecules or simultaneously host two guests [11]. Isaacs and co-workers synthesized two CB[8] derivatives that improved the solubility of various compounds and demonstrated that tetramethyl-CB[8] can act as an antidote for certain drug overdoses [47].

In summary, the cucurbituril family represents a versatile supramolecular platform with exceptional potential for diverse biomedical applications, from diagnostics and targeted delivery to tissue engineering and therapeutic interventions (Table 1).

## 5. Limitations and Future Perspectives

Despite the remarkable structural versatility and biocompatibility of cucurbiturils, several limitations currently restrict their biomedical translation. First, low aqueous solubility, particularly for larger homologues such as CB[6] and CB[8], hampers formulation and in vivo administration. Although derivatization (e.g., perhydroxy- or permethyl-functionalization) can improve solubility, it often increases synthetic complexity and cost [7,24].

Second, data on pharmacokinetics and long-term biodistribution remain limited. Most studies report rapid renal clearance of smaller homologues (CB[5], CB[6]) and slower excretion for CB[7] and CB[8], yet quantitative half-life measurements in plasma are scarce [26,29].

Third, although in vitro and in vivo experiments demonstrate low cytotoxicity and good hemocompatibility, occasional reports of apoptosis, myotoxicity, and cardiotoxicity at higher concentrations indicate the need for comprehensive toxicological profiling before clinical translation [27,28].

Moreover, no clinical trials have been reported so far, and the biodegradability of cucurbiturils remains poorly characterized, raising questions about their long-term persistence in the body and environmental impact [34].

Finally, while perhydroxy-cucurbit[5]uril offers an exciting route toward artificial oxygen carriers, the lack of in vivo validation under physiological or hypoxic conditions still represents a major research gap. Future studies should therefore focus on improving solubility, optimizing functionalization for targeted delivery, and performing detailed pharmacokinetic, toxicological, and efficacy evaluations in animal models prior to any human application.

## 6. Conclusions

Cucurbiturils are recognized as versatile supramolecular hosts with promising biomedical relevance. Functional derivatives, particularly perhydroxy-cucurbit[5]uril, have demonstrated the ability to reversibly bind oxygen under physiological conditions, opening opportunities for the development of synthetic hemoglobin substitutes. In parallel, cucurbituril-based probes for Fe^3+^ and folate provide sensitive diagnostic tools for iron- and folate-deficiency anemia, addressing major global health burdens.

Biocompatibility studies across in vitro, ex vivo, and in vivo models consistently indicate low systemic toxicity and acceptable hemocompatibility for CB[6], CB[7], and CB[8], though isolated reports of apoptosis, myotoxicity, or cardiotoxicity at higher concentrations highlight the need for further toxicological assessment. These results collectively support the use of cucurbiturils as adaptable scaffolds for drug delivery, imaging, and therapeutic modulation.

While translation to the clinic remains preliminary, the structural tunability, stability, and unique recognition properties of cucurbiturils position them as valuable candidates for future applications in oxygen transport and anemia management. Continued interdisciplinary research is essential to validate their safety, optimize formulations, and move toward practical biomedical deployment.

## Figures and Tables

**Table 1:** Representative cucurbituril derivatives, structural modifications, and biomedical applications.

	Cucurbituril/Derivative	Structural Feature/Modification	Biomedical Application	Potential/Perspective	References
1	Perhydroxy-CB[5]	Hydroxyl functionalization	Enhanced oxygen-binding capacity	Potential hemoglobin substitute for O_2_ transport	[3]
2	CB[6] derivatives (mono-/perfunctionalized)	Surface functionalization	Macrocystic vesicles for therapy and bioimaging	Drug delivery, bioimaging, tissue engineering	[34]
3	CB[6] hydrogels	Supramolecular hydrogel structure	3D tissue-mimicking scaffold	Guided chondrogenesis, cartilage regeneration	[44]
4	CB[6] sensor derivative	Selective binding sites	Acetylcholine detection (separated from choline)	Biosensing applications	[45]
5	CB[7]	Native or functionalized macrocycle	Cell targeting, molecular linking, fluorescence imaging, drug delivery	Nanomedicine, gene therapy, molecular machines	[11]
	CB[7]	Supramolecular inclusion of azobenzene dyes; photoisomerizable guest molecules encapsulated in CB[7] cavity	Light-responsive systems (photo-controlled drug release, imaging probes); modulation of azobenzene photophysics by encapsulation	Development of photo-switchable drug carriers, controlled release systems, and optical biosensors; potential in precision medicine and spatiotemporal control of therapy	[48]
6	Fluorescent CB[7]	Fluorescent tagging	Protein localization, membrane fusion monitoring, deep tissue imaging	Advanced bioimaging, diagnostics	[49]
7	CB[8]	Large internal cavity	Binding of large molecules or two guests	Complex formation for supramolecular systems	[11]
8	Tetramethyl-CB[8]	Methyl substitution	Improved solubility of drugs, antidote for overdoses	Detoxification and pharmaceutical solubility enhancement	[47]

## Data Availability

No new data were created or analyzed in this study.
